# Evolution of tomosynthesis

**DOI:** 10.1117/1.JMI.12.S1.S13012

**Published:** 2025-02-12

**Authors:** Mitchell M. Goodsitt, Andrew D. A. Maidment

**Affiliations:** a University of Michigan, Department of Radiology, Ann Arbor, Michigan, United States; b Hospital of the University of Pennsylvania, Department of Radiology, Philadelphia, Pennsylvania, United States

**Keywords:** tomosynthesis, imaging, x-ray

## Abstract

**Purpose:**

Tomosynthesis is a limited-angle multi-projection method that was conceived to address a significant limitation of conventional single-projection x-ray imaging: the overlap of structures in an image. We trace the historical evolution of tomosynthesis.

**Approach:**

Relevant papers are discussed including descriptions of technical advances and clinical applications.

**Results:**

We start with the invention of tomosynthesis by Ziedses des Plantes in the Netherlands and Kaufman in the United States in the mid-1930s and end with our predictions of future technical advances. Some of the other topics that are covered include a respiratory-gated chest tomosynthesis system of the late 1930s, film-based systems of the 1960s and 1970s, coded aperture tomosynthesis, fluoroscopy tomosynthesis, digital detector-based tomosynthesis for imaging the breast and body, orthopedic, dental and radiotherapy applications, optimization of acquisition parameters for breast and body tomosynthesis, reconstruction methods, characteristics of present-day tomosynthesis systems, x-ray tubes, and promising new applications including contrast-enhanced and multimodal breast imaging systems.

**Conclusion:**

Tomosynthesis has had an exciting history that continues today. This should serve as a foundation for other papers in the special issue “Celebrating Digital Tomosynthesis: Past, Present and Future” in the *Journal of Medical Imaging*.

## 1 Birth of Tomosynthesis in the 1930s

### 1.1 Concept

Tomosynthesis was conceived to address a significant limitation of conventional single-projection x-ray imaging: the overlap of structures in an image. This overlap can obscure critical details or create misleading pseudo-objects that resemble pathological conditions. By capturing a series of images at various angles and generating slice images, tomosynthesis significantly reduces this superposition. Unlike computed tomography (CT), which acquires high-resolution images over a 360-deg range, tomosynthesis collects significantly higher resolution projections over a limited angle range (e.g., 10 to 60 deg). The resulting tomosynthesis images exhibit higher in-plane spatial resolution compared with CT but lower resolution in the depth axis.

**Fig. 1:**
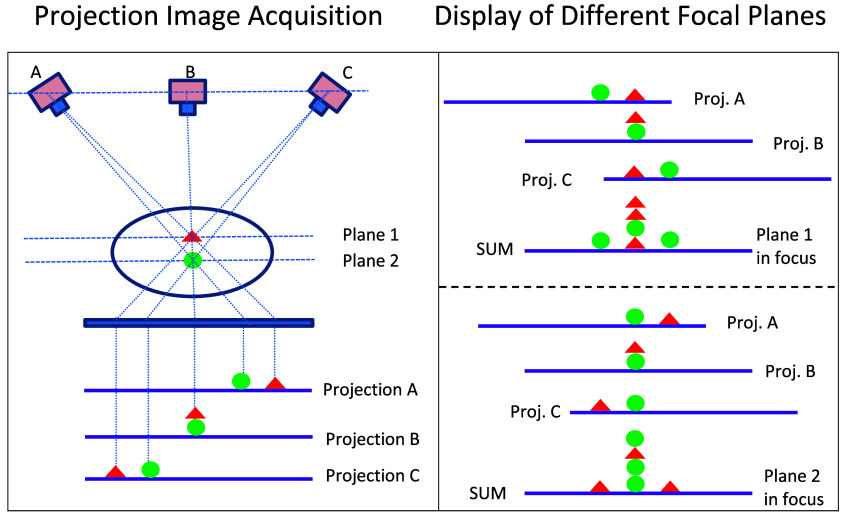
Illustration of tomosynthesis projection image (film) acquisition from different angles (left) and the method of shifting and adding those images to display different focal planes within the body (right). Note that the tomosynthesis methods are distinct from film tomography in which the x-ray source and detector (initially film) are moved in opposition about a fulcrum; the film tomography methods yield a single slice image per acquisition. Today, modern digital tomosynthesis systems employ digital radiographs and sophisticated reconstruction algorithms akin to those used in CT.

In early implementations, a series of film radiographs were captured from different angles as the x-ray tube was moved in steps across the patient. These images were then aligned and combined, or “shifted and added,” to focus the readers’ attention on different planes within the body, as illustrated in Fig. 1.

### 1.2 Inventors—Ziedses des Plantes and Kaufman

Bernard George Ziedses des Plantes, a Dutch neuroradiologist and electrical engineer, is often credited with inventing tomosynthesis. His pioneering work in the 1930s laid the foundation for this innovative imaging technique. Ziedses des Plantes first published an article about the technique, which he called “seriescopy,” in 1935.^1^ This technique aimed to overcome the limitations of conventional radiography by allowing for the visualization of specific planes within the body, thus reducing the problem of superimposed structures in single projection images. In 1936, he patented seriescopy, solidifying his role as a foundational figure in the development of tomosynthesis.^2^

In a 1938 paper, Ziedses des Plantes described seriescopy as a “radiographic method which makes it possible to view an infinite series of parallel planes in succession by means of a few exposures.”^3^ This method involved a prototype system based on circular tomography. The system took four stationary film exposures at equidistant points along the circular path of the x-ray tube, typically at 0 deg, 90 deg, 180 deg, and 270 deg. To achieve different planes of focus, the developed films were reciprocally moved above a single viewing screen. In addition, Ziedses des Plantes proposed an optical reconstruction method using mirrors to superimpose the images of the films and the rocking of the mirrors to achieve the same effect as translating the films.^4^ He also described a device for recording the level of the plane of focus, which added another layer of precision to the technique.^4^

Julius Kaufman, a medical doctor from Brooklyn, New York, independently developed a similar method called “planeography” in 1936.^5^ Similar to Ziedses des Plantes, Kaufman sought to enhance radiographic imaging by isolating specific planes within the body. His planeography technique made it possible to demonstrate any plane in space parallel to the film plane using two or more properly taken radiographs. Kaufman emphasized the method’s ability to localize structures and measure depth, using a “standard depth curve” to relate the height of a linear object in a plane to the spread of its projections in the films.

Although Kaufman’s work was contemporaneous with that of Ziedses des Plantes, and despite the similarities in their concepts, Kaufman is less frequently recognized in the context of tomosynthesis. Nonetheless, his contributions were significant. Kaufman published several papers detailing the applications of planeography^6^^,^^7^ and collaborated with Harry Koster, MD, to critically analyze and compare their technique with Ziedses des Plantes’ seriescopy and Paul Cottenot’s serioscopy.^8^ They highlighted issues such as unequal magnification and provided solutions to correct these problems.

Given the similarities between their inventions and the fact that both appear to have worked independently without knowledge of each other’s efforts, it is fair to credit both Ziedses des Plantes and Kaufman with the invention of tomosynthesis. Their parallel developments laid a robust foundation for the evolution of this essential imaging technology, marking a significant milestone in the history of medical radiography.

### 1.3 Cottenot’s Early Chest Tomosynthesis System

In 1937, French radiologist Paul Cottenot presented an influential paper at the Fifth International Congress of Radiology in Chicago, Illinois. In his presentation, Cottenot described an innovative method for tomosynthesis, specifically aimed at studying pleuro-pulmonary lesions. He termed this technique “Thoracic Serioscopy.”^9^ Cottenot’s work was notably influenced by the earlier seriescopy method developed by Ziedses des Plantes.

Cottenot’s system was designed to address a critical challenge in tomosynthesis chest imaging, ensuring that each projection radiograph was taken at the same level of inspiration. This consistency was crucial for accurate tomosynthesis imaging of the chest. To achieve this, Cottenot developed a “respiratory trigger” mechanism. The imaging procedure involved capturing four exposures with the x-ray tube positioned 14 cm to the left, right, upward, and downward of the center. Each exposure was set to approximately one-third the duration of a conventional radiograph to minimize motion blur. This approach ensured that the x-ray images were taken at consistent and controlled inspiration levels, thereby improving the accuracy of the tomosynthesis process.

After the films were developed, they were viewed in a specially designed high-intensity light box. This viewing system featured screws that allowed for precise shifting of the films relative to each other. The viewing apparatus included a pointer and a graduated dial marked in centimeters, which facilitated the measurement of different planes within the chest.

Cottenot’s thoracic serioscopy system proved to be effective clinically. He successfully demonstrated its application in determining the dimensions and locations of pneumothorax, lung abscesses, and tuberculosis foci.^9^ His work laid important groundwork for the development of more advanced tomosynthesis systems and highlighted the potential of this technology in improving diagnostic accuracy for chest diseases.

## 2 Film-Based Systems of the 1960s and 1970s

### 2.1 Developments by Garrison and Grant

Tomosynthesis saw renewed interest in the late 1960s after a hiatus that started in the late 1930s when film tomography became dominant. John Garrison and David Grant at Johns Hopkins University developed a prototype involving a circular scan capturing twenty radiographs.^10^ These images were photo-reduced and reprojected using an optical back-projection system with mirrors. Grant coined the term “tomosynthesis” in a 1972 paper, marking a significant milestone in the technology’s development.^11^ In this seminal paper, Grant discussed both film and digital methods for acquiring tomosynthesis images.

### 2.2 Rapid Film Changer Tomosynthesis Systems

#### 2.2.1 Dynatome system by Albert Richards

In the 1970s and 1980s, Albert Richards, a physicist and professor of dentistry at the University of Michigan Dental School, developed the first commercial film-based tomosynthesis system known as the Dynatome. This innovative system was designed to provide detailed cross-sectional images of the body by utilizing a rapid film changer mechanism to capture multiple radiographs at different angles.

**Fig. 2:**
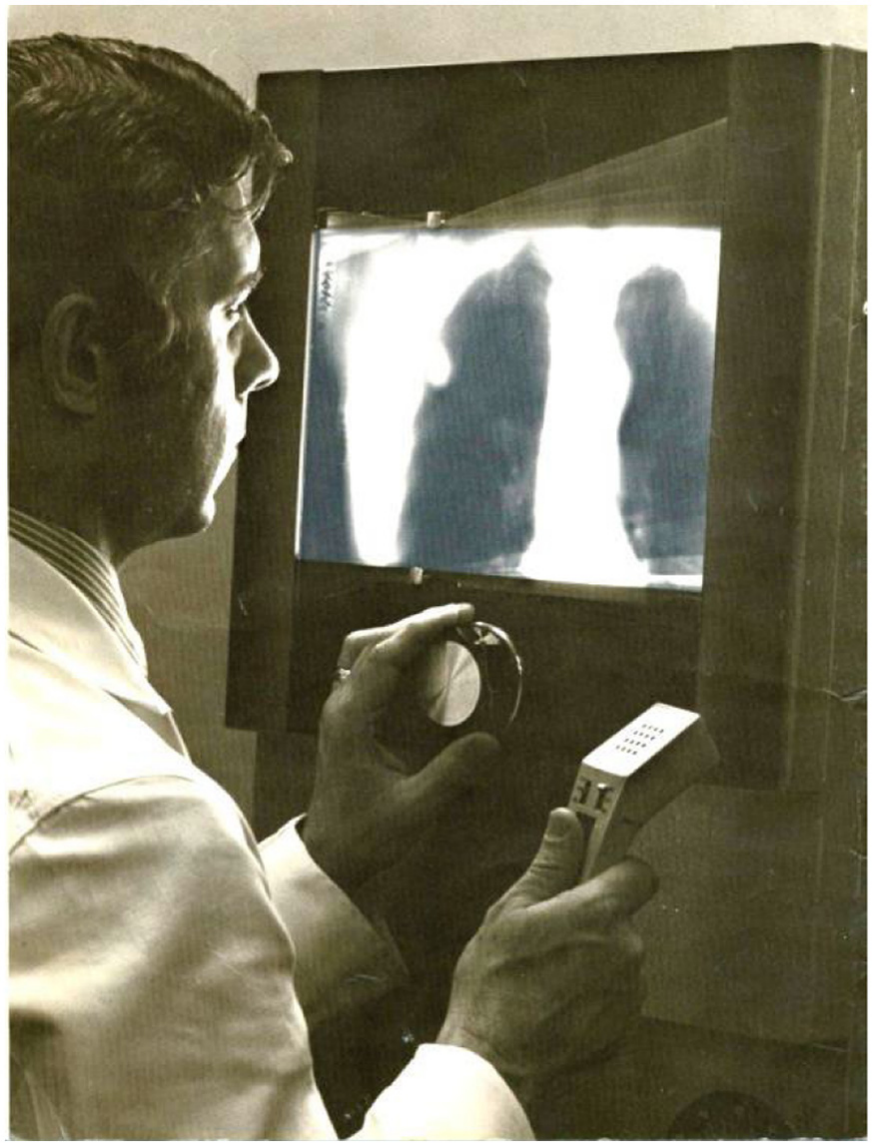
Custom high-intensity light box film viewer for Dynatome tomosynthesis system.

A crucial component of the Dynatome system was the calibrator, which determined the precise angle for each film exposure. This calibrator ensured that the films were trimmed and shaped correctly based on their projection angles. After the films were developed, they were arranged on a custom high-intensity light box (see Fig. 2). This viewer was equipped with a precise mechanical system, allowing the operator to move the films relative to each other using a dial. This precise movement enabled the registration of information from any level common to all films, effectively creating contiguous tomographic slices.

The system allowed for the viewing of ∼100 contiguous slices, each with an effective thickness of 2 mm. This capability provided a detailed cross-sectional view of the body, enhancing the detection and diagnosis of various medical conditions. Despite the multiple exposures required, the total radiation dose to the patient was roughly equivalent to that of two or three standard plain radiographs.

Richards described the principles and advantages of dynamic tomography, his term for tomosynthesis, in a paper published in 1976.^12^ In this paper, he explained that moving the x-ray source in a circular path yielded better results than linear movements. Richards also foresaw future applications of dynamic tomography, such as imaging the beating heart layer by layer, which he predicted could be achieved with electrocardiogram (EKG) gating and very short exposures.^12^

### 2.3 Coded Aperture Tomosynthesis

During the 1970s, one of the most advanced tomosynthesis methods of the era was developed by Klotz and Weiss at Philips GmbH in Hamburg, Germany.^13^^–^^16^ This technique, known as “Flashing Tomosynthesis,” allowed for the generation of tomosynthesis planes in mere milliseconds, which effectively eliminated issues related to patient motion.

The Flashing Tomosynthesis method utilized multiple small stationary x-ray sources, which were arranged in a fixed configuration around the patient. Instead of moving a single x-ray source, these multiple sources were pulsed either sequentially or simultaneously. A single large film was used to record the entire set of sub-images, resulting in a coded image that required a specialized process for reconstruction.

The term “coded-aperture imaging” refers to this technique’s unique approach to capture and reconstruct images. To decode the image, Klotz and Weiss devised an optical reconstruction method, in which the coded image was illuminated on a light box and viewed through an array of lenses arranged according to the distribution of the x-ray tubes. By positioning a ground glass screen at the appropriate depth within the 3D image, different layers of the object could be brought into focus and visualized.

The system utilized twenty-four small stationary x-ray tubes arranged in a circular pattern around the patient. These tubes were fired simultaneously, with an extremely short exposure time of about 50 ms. This rapid exposure significantly reduced the likelihood of motion artifacts, resulting in clearer images.^17^ The images produced by this “Flashing Tomosynthesis” system had an effective slice thickness of about 1 mm, providing high-resolution cross-sectional views. Despite its advantages, the system had some limitations, such as a small field of view with a diameter of only 9 cm. In addition, the system was not capable of evaluating blood flow, as it was limited to a single “flash” exposure for each tomosynthesis image.^17^

Additional studies and publications on flashing and coded aperture tomosynthesis have been conducted by various researchers, building on the principles established by Klotz and Weiss. Some of these include the works of Groh,^18^ Nadjmi et al.,^19^ Stietl et al.,^20^^–^^22^ Haaker et al.,^23^^–^^25^ and Becher et al.^26^^,^^27^

## 3 Fluoroscopic Tomosynthesis

Fluoroscopic applications of tomosynthesis emerged in the early 1970s, developed by two separate groups: one led by Bailey et al. at the University of California, San Diego (UCSD),^28^^–^^30^ and the other by Hoeffner et al.^31^ at Philips GmbH in Hamburg, Germany. These pioneering efforts aimed to enhance real-time imaging capabilities by integrating the principles of tomosynthesis with fluoroscopy, thus enabling dynamic, multi-plane visualization.

### 3.1 System by Bailey et al. at UCSD

Bailey and his colleagues at UCSD designed an advanced fluoroscopic tomosynthesis system that combined a pulsed x-ray source with an image intensifier and TV camera detector. The system was designed to capture high frame rates and provide good contrast at relatively low radiation doses, making it suitable for dynamic imaging applications.

The UCSD system consisted of four primary components:

1. a linear tomography device with a pulsed x-ray tube that moved along a predefined path
2. an image intensifier and TV camera that was synchronized with the image acquisition
3. a video disk recorder that stored the projection images
4. a minicomputer for reconstruction which calculated the tomosynthesis slices

The UCSD system allowed for dynamic assessment of anatomical structures, providing valuable real-time insights that were not possible with static imaging techniques. This made it particularly useful for applications requiring continuous observation, such as interventional procedures and motion studies.

### 3.2 System by Hoeffner et al. at Philips

Concurrently, Hoeffner and his team at Philips GmbH developed a similar fluoroscopic tomosynthesis system. Their approach also utilized an image intensifier and TV camera detector, along with a system to capture and reconstruct multiple projection images in real-time.

The Philips system used a pulsed x-ray source and image intensifier like the UCSD system. It provided both analog and digital storage solutions. The Philips system also supported both linear and circular tomography, using advanced digital reconstruction algorithms.

#### 3.2.1 Further Developments in Fluoroscopic Tomosynthesis

Building on the foundational work of these early systems, further developments in fluoroscopic tomosynthesis focused on improving image quality, reducing radiation dose, and enhancing computational reconstruction techniques. Digitization of the video signal allowed for more precise image manipulation and storage.

Researchers at institutions such as the University of Utah (Kruger,^32^^,^^33^ Anderson,^34^ and de Vries^35^) and the University of Texas (Maravilla,^36^^–^^38^ Murry and Maravilla^39^) explored the potential of tomosynthesis for digital subtraction angiography (DSA) applications. By combining tomosynthesis with contrast media, they aimed to enhance the visualization of blood vessels, providing clearer images of vascular structures. This approach, known as DSA tomosynthesis, allowed for the subtraction of background tissue, highlighting the contrast-filled vessels with greater clarity.

## 4 Clinical Applications of Fluoroscopic Tomosynthesis

Fluoroscopic tomosynthesis has been applied in various clinical settings, providing real-time 3D imaging for procedures such as interventional radiology (Alhrishy^40^), cardiac imaging (Spiedel^41^), and pulmonary imaging (Pritchett,^42^ Gmehlin,^43^ Jain^44^).

Overall, the integration of tomosynthesis with fluoroscopic imaging significantly enhances the capabilities of real-time medical imaging, offering detailed, multi-plane visualization that improves diagnostic accuracy and procedural outcomes.

## 5 Digital Detector-Based Tomosynthesis

In the late 1990s, the introduction of flat-panel digital detectors revolutionized tomosynthesis by providing superior spatial resolution and sensitivity compared with traditional film-based systems. These detectors converted x-ray photons into electrical signals with high efficiency. This enhanced conversion capability allowed for clearer and more detailed images at lower radiation doses, making digital tomosynthesis a more viable and safer option for patients. The high detective quantum efficiency (DQE) of these detectors was particularly beneficial for applications requiring detailed visualization of fine structures, such as breast tissues and small lung nodules. Flat-panel detectors also facilitated the integration of tomosynthesis with other imaging modalities, such as CT and MRI, expanding the clinical applications of the technology.

Digital detectors also provided the ability to process and manipulate images digitally, facilitating advanced image reconstruction algorithms that further improved image quality and diagnostic accuracy. Furthermore, the transition to digital detectors also brought about significant improvements in workflow and storage. Digital images could be stored, retrieved, and transmitted electronically, streamlining the imaging process and enhancing the ability to share and review images remotely.

Early pioneers of digital detector-based tomosynthesis, such as Niklason and Kopans at Massachusetts General Hospital, collaborated with industry partners such as General Electric (GE) to develop prototype systems for full-field breast imaging.^45^ These systems demonstrated the potential of digital tomosynthesis to improve lesion visibility, particularly in dense breast tissue, by providing three-dimensional reconstructions that mitigated the issue of tissue overlap inherent in traditional mammography.

Simultaneously, researchers such as Dobbins et al.^46^^–^^48^ at Duke University explored the application of digital tomosynthesis in chest imaging. Their work demonstrated significant improvements in the detection of lung nodules compared with conventional chest radiography, highlighting the potential of digital tomosynthesis to enhance the diagnosis of pulmonary diseases.

The shift to digital detector-based tomosynthesis set the stage for further innovations and applications across various medical fields, from orthopedics to cardiology, solidifying its role as a critical tool in modern diagnostic imaging.

### 5.1 Digital Detector Breast Imaging Systems

#### 5.1.1 System developed by Niklason, Kopans, and GE

Loren Niklason and Daniel Kopans at Massachusetts General Hospital, in collaboration with scientists at GE, were among the early pioneers in developing a digital detector-based tomosynthesis system for breast imaging, which is often termed digital breast tomosynthesis (DBT). This innovative system was based on the GE DMR film/screen mammography unit, which was modified to incorporate a stationary flat-panel digital detector.

The flat-panel detector used in this system was constructed with a cesium iodide (CsI) scintillator coupled to an amorphous silicon (a-Si) transistor-photodiode array. This combination was chosen for its high DQE and excellent spatial resolution. The detector featured a pixel pitch of 100  μm, allowing for the capture of fine details in breast tissue. The readout time for the detector was approximately 300 ms, enabling the rapid acquisition of multiple images needed for tomosynthesis.

To perform tomosynthesis, the x-ray source moved along an arc, capturing images at different angles. The system typically acquired nine equally spaced projection images over a 40-deg tomosynthesis angle. This range and number of images were found to provide an optimal balance between image quality and radiation dose. The resulting projection images were then reconstructed into tomosynthesis slices using advanced algorithms, which allowed for the visualization of breast tissue in three dimensions.

In preliminary studies using breast phantoms and cadaveric breast specimens, the Niklason-Kopans system demonstrated a significant improvement in lesion visibility compared with traditional two-dimensional mammography. The three-dimensional tomosynthesis reconstructions reduced tissue overlap, which can obscure lesions in conventional mammography. This ability was particularly beneficial for women with dense breasts, where the presence of overlapping normal tissue structures can mask the presence of tumors.^45^

One of the key findings from these early studies was that tomosynthesis could enhance the specificity of mammography. By providing clearer images of lesion margins, tomosynthesis helps differentiate between benign and malignant lesions more effectively. This improved specificity had the potential to reduce the number of false-positive results and negative biopsies, thereby improving patient care and reducing healthcare costs.

The collaboration between Massachusetts General Hospital and GE laid the groundwork for the development of commercial DBT systems. Their prototype system demonstrated the feasibility and advantages of tomosynthesis in clinical breast imaging, paving the way for further advancements and widespread adoption of this technology in breast cancer screening and diagnosis.

#### 5.1.2 Tuned aperture CT digital spot tomosynthesis—Webber and instrumentarium

Prior to the widespread availability of large-area flat-panel digital detectors, Richard Webber developed a “spot” tomosynthesis device that imaged a limited region of the body using a small-area digital detector. Webber’s device used a novel tomosynthesis methodology which he called “tuned aperture computed tomography” (TACT).^49^^–^^55^ TACT represented a significant innovation in the field of digital spot tomosynthesis, particularly for dental and later breast imaging applications.^50^^–^^55^ TACT was initially designed for dental imaging to provide detailed views of teeth and surrounding structures. This technology utilized a unique approach to reconstruct images from multiple x-ray projections taken at arbitrary angles and orientations.

The TACT system involves capturing multiple digital radiographic images from different angles around the area of interest. Unlike traditional tomosynthesis, which requires a fixed and predetermined set of angles, TACT allows for flexibility in image acquisition, making it easier to adapt to various clinical scenarios. The key to TACT’s effectiveness is the use of fiducial markers placed in or near the area being imaged. These markers serve as reference points for accurately aligning and reconstructing the images.

After the images are captured, they are processed using specialized software that aligns the images based on the fiducial markers. The software then calculates the center of gravity for each point of interest, allowing for precise shifts of the images relative to one another. By stacking and averaging the superimposed pixels, the system generates high-resolution tomographic slices that reveal detailed structures within the imaged volume.

Instrumentarium (Helsinki, Finland) recognized the potential of TACT for broader medical applications and licensed the technology from Webber. They developed the Delta 32 TACT^®^ system, which was introduced to the market at the Radiological Society of North America meeting in 1997 and received FDA approval in 2000. This system was specifically designed for breast imaging, adapting the principles of TACT to provide clearer views of breast tissue and improve lesion detection.

The Delta 32 TACT^®^ system used a small 5×5  cm charge-coupled device detector. During the imaging process, seven projections were acquired at different angles around the breast. The images were then digitally reconstructed to produce tomographic slices, providing a detailed three-dimensional view of the breast tissue. This approach was particularly beneficial for spot imaging of specific areas of concern, such as small lesions or microcalcifications.

In clinical studies, the Delta 32 TACT^®^ system showed promise in improving the detection and characterization of breast lesions. By providing high-resolution images with reduced tissue overlap, TACT helped radiologists more accurately differentiate between benign and malignant findings. This capability was crucial for early detection and effective treatment planning in breast cancer.

Today, several manufacturers are marketing DBT biopsy systems. These use small-area flat-panel digital detectors for prone geometry units and large-area flat-panel digital detectors for upright geometry units. They employ more conventional types of DBT acquisition and reconstruction than TACT.

#### 5.1.3 Other early flat-panel digital tomosynthesis breast imaging systems

Other pioneers in the development of flat-panel digital tomosynthesis for breast imaging included Suryanarayanan and Karellas at the University of Massachusetts, Worcester.^56^^,^^57^ Their system, similar to that of Niklason and Kopans, employed a GE 2000 D digital mammography unit. However, instead of using a 40-deg tomosynthesis angle with nine 5-deg increments, they opted for a 36-deg angle with seven 6-deg increments. They also investigated various tomosynthesis reconstruction algorithms, including TACT backprojection, TACT maximization, TACT minimization, TACT iterative restoration, expectation maximization, and Bayesian smoothing iterative methods.^56^^,^^57^

#### 5.1.4 Optimization of acquisition parameters in breast tomosynthesis

Several research groups have focused on optimizing acquisition parameters such as total sweep angle, angle increment, and radiation dose for breast tomosynthesis. Studies have explored different configurations to enhance image quality and reduce radiation exposure, ensuring the best possible diagnostic outcomes. Some relevant papers on this topic have been published (Wu et al.,^58^ Godfrey et al.,^59^ Deller et al.,^60^ Zhao et al.,^61^ Zhou et al.,^62^ Gifford et al.,^63^ Reiser et al.,^64^ Chawla et al.,^65^ Chan et al.,^66^^,^^67^ Goodsitt et al.,^68^ Sechopoulos and Ghetti,^69^ Chawla et al.,^70^ Marshall, and Bosmans^71^). Highlights include the use of computer simulation,^62^ model observers,^63^^,^^64^ experimental studies of phantoms with a versatile DBT prototype that permitted the use of a wide range of acquisition parameters,^67^^,^^68^ a combination of variable sweep angle/increment experimental imaging of mastectomy specimens with insertion of simulated 3D lesions and a model observer,^70^ and virtual clinical trials.^71^ In general, the results tend to show that there is improved perception of masses with wide-angle acquisitions and improved detection of microcalcifications with small-angle acquisitions.

### 5.2 Digital Tomosynthesis for Body Imaging

#### 5.2.1 Chest imaging

The development and application of digital tomosynthesis in chest imaging have demonstrated significant advancements, primarily led by researchers such as Dobbins and colleagues at Duke University. Their work has been pivotal in showcasing the potential of tomosynthesis to improve the detection and diagnosis of lung diseases compared with conventional chest radiography.

The prototype system developed by Dobbins et al. at Duke University utilized a state-of-the-art GE flat-panel detector specifically designed for chest imaging (Dobbins and McAdams^48^). This detector featured a large field of view, measuring 41 cm by 41 cm, with a cesium iodide (CsI) scintillator coupled to an amorphous silicon (a-Si) photodiode array. This combination provided high DQE and excellent spatial resolution, with a pixel pitch of 200  μm, enabling the capture of fine details in chest anatomy.

The imaging process involved a pulsed x-ray source moving in a vertical trajectory during the scan, capturing multiple projection images at different angles. The optimized technique for this prototype system consisted of 71 projections over a 20-deg angular range. This acquisition strategy provided a balance between image resolution and radiation dose, ensuring optimal image quality for clinical diagnosis.

The reconstruction of tomosynthesis slices was performed using an algorithm known as matrix inversion tomosynthesis (MITS). This algorithm applied principles of linear algebra to correct for out-of-plane blur, enhancing the clarity of in-plane structures. The MITS algorithm processed the projection data to generate a series of contiguous tomographic slices, each separated by 5 mm providing detailed cross-sectional views of the chest.

Initial clinical trials conducted at Duke University demonstrated the superior performance of digital tomosynthesis in detecting lung nodules compared with conventional chest radiography. The study involved patients undergoing both conventional radiographic and tomosynthesis imaging, with radiologists assessing the images for the presence of lung nodules. The results showed that tomosynthesis significantly improved the sensitivity of nodule detection. The 3D capability of tomosynthesis allowed radiologists to visualize nodules with greater depth perception, reducing the issue of overlapping structures that often obscured nodules in traditional 2D radiographs.

Following these promising results, similar studies were conducted at other institutions, such as the University of Gothenburg using the GE VolumeRad system, a commercial digital chest tomosynthesis system. These studies further validated the clinical benefits of tomosynthesis, showing that it could detect up to three times as many lung nodules as conventional chest radiography (Vikrgren^72^).

The application of digital tomosynthesis in chest imaging extends beyond nodule detection. It has been utilized for evaluating various pulmonary conditions, including interstitial lung disease, pneumonia, and pleural effusions. The ability to visualize different layers of lung parenchyma in fine detail aids in the assessment of disease extent and progression, providing valuable information for treatment planning and monitoring (Rimkus et al.,^73^ Sone et al.,^74^^–^^76^ Matsuo et al.,^77^ Dobbins et al.,^46^ Godfrey et al.,^78^^–^^80^ Fahrig et al.,^81^ Yamada et al.,^82^ Asplund et al.,^83^ Gomi et al.,^84^ Kim et al.,^85^ Quaia et al.,^86^^,^^87^ Johnsson et al.,^88^ Santoro et al.,^89^ Zachrisson et al.,^90^ and Jung^91^).

Moreover, digital tomosynthesis has been explored for use in low-dose screening programs, particularly for lung cancer. Given its lower radiation dose compared with CT and its higher diagnostic accuracy compared with traditional radiography, tomosynthesis presents a viable option for large-scale screening initiatives aimed at early detection of lung cancer, potentially improving patient outcomes through earlier intervention.

In summary, the development and application of digital tomosynthesis in chest imaging represents a significant advancement in radiology. The work by Dobbins et al. and subsequent clinical validations have established tomosynthesis as a powerful tool for the detection, diagnosis, and management of pulmonary diseases.

#### 5.2.2 Orthopedic, dental, radiotherapy, and other applications of tomosynthesis

Tomosynthesis has found a variety of applications beyond breast and chest imaging, significantly impacting fields such as orthopedics, dental imaging, and radiotherapy. These diverse applications leverage the unique ability of tomosynthesis to provide detailed, cross-sectional views of complex structures, enhancing diagnostic accuracy and treatment planning.

##### Orthopedic imaging

In orthopedics, tomosynthesis is particularly useful for imaging joints (Rinkus,^73^ Sone,^74^ Kolistri,^92^ Duryea,^93^^,^^94^ Fahey,^95^ Gazaille;^96^ fractures, Mermuys^97^ and Geijer^98^) and implanted hardware (Gomi^99^). For example, in joint imaging, tomosynthesis provides clear views of the joint space and surrounding bone structures, aiding in the diagnosis of conditions such as osteoarthritis and rheumatoid arthritis. It is also valuable in assessing the alignment and integrity of fractures, offering a more comprehensive evaluation than conventional x-rays. In addition, tomosynthesis reduces metal artifacts commonly associated with orthopedic implants, improving visualization of bone-implant interfaces and potential complications such as loosening or infection.

##### Dental imaging

In dental imaging, tomosynthesis is used to obtain high-resolution images of teeth and jaw structures. It provides detailed views for the assessment of dental caries, periodontal disease, and other oral health issues. The ability to capture images at various angles and reconstruct them into 3D slices allows for precise localization of dental lesions and abnormalities, which is particularly beneficial for endodontic and orthodontic evaluations (Richards,^12^ Groenhuis,^100^ Ruttiman,^101^^,^^102^ van der Stelt,^103^^,^^104^ Engelke,^105^ Vandre,^106^ Webber,^50^^–^^53^ Horton,^107^ Tyndall,^108^ Lauritsch and Harer,^109^ Nair,^110^^,^^111^ Abreu,^112^ Gomi,^113^ Ogawa,^114^ and Katsumata^115^).

##### Radiotherapy planning and verification

Tomosynthesis has significant applications in radiotherapy, particularly in treatment planning and verification. Accurate imaging is crucial for precisely targeting tumors while sparing surrounding healthy tissue. Tomosynthesis provides detailed, volumetric data that can be integrated into radiotherapy planning systems to improve the accuracy of dose delivery.

Tomosynthesis is used for image-guided radiation therapy. By capturing real-time images of the treatment area, clinicians can verify the patient’s position and make necessary adjustments to ensure the radiation beams are accurately focused on the tumor. This capability enhances the precision of treatments such as intensity-modulated radiation therapy and stereotactic body radiotherapy, leading to better clinical outcomes (Zwicker and Atari,^116^ Messaris,^117^ Persons,^118^ Tutar,^119^ Godfrey,^59^^,^^120^ Yan,^121^ Wu,^122^ Ren,^123^ Pang,^124^ Maurer,^125^^,^^126^ Sarkar,^127^ Yoo,^128^ Zhang,^129^ Maltz,^130^ Mestrovic,^131^ Brunet-Benkhoucha,^132^ Lyatskaya,^133^ Winey,^134^^,^^135^ Park,^136^^,^^137^ and Wu^138^).

##### Other clinical applications

Tomosynthesis has also been explored for various other clinical applications, including

• **Inner ear imaging**: Tomosynthesis provides detailed images of the inner ear structures, aiding in the diagnosis of conditions such as cholesteatoma and otosclerosis. The ability to visualize the bony labyrinth in fine detail helps in preoperative planning and postoperative assessment. (Chakraborty,^138^ Sone^74^).
• **Abdominal imaging**: Although CT remains the gold standard for abdominal imaging (Nelson,^139^ Sone^74^), tomosynthesis has been used to evaluate the kidney (Mermuys^140^). The technique offers a lower radiation dose alternative with sufficient detail for specific diagnostic needs.
• **Knee imaging**: Tomosynthesis is valuable for assessing soft tissue structures around the knee, including ligaments and menisci. It provides a detailed view of these structures, facilitating the diagnosis of tears and other injuries (Rimkus,^73^ Sone,^74^ and Flynn^141^).

#### 5.2.3 Optimizing parameters in body tomosynthesis

Machida et al.^142^ published a comprehensive tutorial on optimizing parameters in digital body tomosynthesis. The paper discusses factors such as sweep angle, sweep direction, patient barrier-object distance, the number of projections, and total radiation dose. It also addresses acquisition-related artifacts such as blurring, ripple, and ghosting, offering strategies to minimize these issues and improve image quality.

## 6 Brief History of Tomosynthesis Reconstruction Methods

Tomosynthesis reconstruction methods have evolved from simple “shift and add” techniques to sophisticated iterative methods and deep learning approaches. Early systems employed unfiltered backprojection, whereas modern systems utilized advanced algorithms to enhance image quality and reduce artifacts.

### 6.1 Shift and Add

The shift and add method, used since 1935, brings in-plane objects into focus while blurring out-of-plane features and can be considered unfiltered backprojection (Machida^142^). This basic technique has been foundational in tomosynthesis imaging. The shift and add method approximates the process of back-projecting the source images to a common plane and then sums the unfiltered images. The result amplifies structures contained within the focal/reconstruction plane while diminishing the conspicuity of objects further from the reconstruction plane.

### 6.2 Matrix Inversion Tomosynthesis

MITS, developed by Dobbins et al., uses linear algebra to correct for out-of-plane blur, providing clearer reconstructions. This method has been in use since 1987 (Dobbins,^143^^,^^144^ Godfrey,^59^^,^^78^^–^^80^^,^^145^ and Warp^146^). More recent advances in the method blended the linear algebra reconstructions with filtered backprojection reconstructions, based upon a spatial frequency threshold. This method led to more stable results and better-quality images.

### 6.3 Filtered Backprojection

Filtered backprojection, the most common CT reconstruction method, applies low-pass filters to suppress high frequencies and compensate for incomplete or non-uniform sampling. This technique has been adapted for tomosynthesis since 1998 (Lauritsch and Harer,^109^ Badea,^147^ and Stevens^148^). It is the most commonly used method for image reconstruction and still forms the basis for most commercial products, although most products add pre- and post-processing to ameliorate artifacts in the reconstructions, for example, to deal with highly attenuating structures in images.

### 6.4 Iterative Reconstruction Techniques

Algebraic reconstruction techniques (ARTs), used in the first commercial CT scanners, solve a set of linear equations iteratively (Gordon^149^ and Meyer-Ebrecht and Wagner^150^). Variants such as simultaneous ART (Andersen and Kak^151^) and simultaneous iterative reconstruction technique have been developed (Colsher^152^).

Statistical reconstruction methods, such as maximum likelihood (ML) and its variants ML-EM (Lange and Fessler^153^) and ML-convex (Lange,^154^ Lange and Fessler,^153^ Wu^58^^,^^155^), determine the 3D model of x-ray attenuation coefficients that maximize the probability of obtaining the measured projections. These methods have been in use since the 1990s.

Other iterative reconstruction methods including model-based iterative reconstruction have also been researched and used commercially.

### 6.5 Deep Learning Reconstruction

In recent years, deep convolutional neural network (CNN) methods have been increasingly applied to the reconstruction of breast and body tomosynthesis images, yielding significant advancements. The primary objectives of these methods include:

• **Restoring full Data from sparse data**: For low-dose chest tomosynthesis, CNN techniques have been employed to reconstruct full data from sparse datasets, effectively reducing radiation exposure while maintaining image quality (Lee and Kim^156^).
• **Noise reduction and texture preservation**: In DBT, CNN methods aim to reduce noise while preserving background texture, ensuring that subtle microcalcifications remain visible without blurring (Gao^157^).
• **Improving contrast and in-depth resolution**: Enhancing contrast and in-depth resolution in DBT images is another goal, leading to clearer and more detailed visualizations of breast tissue (Wu^158^).
• **Enhancing spatial resolution**: For chest tomosynthesis, CNN-based reconstruction methods have been shown to improve spatial resolution, providing more precise and detailed images (Kim^159^).
• **Accurate breast density and dose estimation**: Deep learning techniques have also been applied to improve the accuracy of breast density assessments and patient-specific radiation dose estimations, which are critical for personalized care (Teuwen^160^).
• **Enhancing lung therapy guidance**: In the context of chest tomosynthesis, deep learning methods have been utilized to enhance image quality for better guidance during lung therapy (Jiang^161^).

## 7 Methods to Reduce Blur From Out-of-Plane Details

Various methods have been developed to address the issue of blur from out-of-plane objects in tomosynthesis reconstructions. Techniques such as unsharp masking (Edholm and Quiding^162^^,^^163^), high-pass frequency filtering (Chakraborty,^164^ van der Stelt et al.,^103^ and Sone,^76^ Lu^165^), and wavelet-based methods (Badea^166^) have been employed to enhance in-plane contrast. MITS and other advanced algorithms have further improved the clarity and accuracy of tomosynthesis images (Dobbins and Godfrey^167^).

## 8 Present-Day Tomosynthesis Systems

Numerous body tomosynthesis systems have been developed over the years, but only a few have received FDA approval. In 2006, GE Healthcare was the first company to receive FDA approval for their VolumeRad tomosynthesis system, which was designed for body imaging (chest, knee, legs, etc.), and Shimadzu followed in 2008, receiving FDA approval for their Sonialvision Safire II body tomosynthesis system.

The first FDA approval for a DBT system came on February 11, 2011, when Hologic received approval for their Selenia Dimensions system. Since then, several other DBT systems have also gained FDA approval. These include the Hologic 3Dimensions, the GE SenoClaire, the GE Senographe Pristina, the Siemens Mammomat Inspiration, and the Fujifilm ASPIRE Cristalle (marketed as the Amulet Innovality outside the United States). Most recently, in 2024, the FDA approved the Siemens B.brilliant DBT system. That system is unique in that it uses a flying focal spot technology to keep the focal spot location stationary during exposures while the x-ray tube moves continuously. Additional systems are available outside the United States. A brief summary of some of the features of present-day digital breast imaging systems is provided in Table 1.

**Table 1:** Some characteristics of present-day DBT systems.

Unit	Tomo angle (deg)	# views	Pixel pitch/size (μm)^a^	2 × 2 binning^b^	Detector	Scan time (s)
Fujifilm Amulet Innovality	15 (ST)^c^	15 (ST)	68/100 to 150 (ST)^c^	Yes	a-Se	3.5 (ST)
Fujifilm Amulet Innovality	40 (HR)^c^	15 (HR)	68/50 to 100 (HR))^c^	No/Yes	a-Se	9.4 (HR)
GE Pristina	25	9	100/100	No	CsI-a-Si	9
Hologic Selenia Dimensions	15	15	70/95 to 117	Yes	a-Se	3.7
Hologic 3Dimensions	15	15	70/70	No	a-Se	3.7
IMS Giotto Class	30	11	85/90	No	a-Se	9.2
Planmed Clarity	30	15	83/95	No	a-Se	18
Siemens Mammomat Inspiration	50	25	85/85	No	a-Se	21
Siemens Mammomat B.brilliant	50	25	85/85	No	a-Se	5

a Pixel size in the focal plane.

b Binning involves combining information from adjacent pixels. For example, the first Hologic system combined 2×2 blocks of 70-μm pixels to create 140-μm “binned” pixels.

c ST, standard mode; HR, high-resolution mode.

In a recent paper by Marshall and Bosmans (Marshall and Bosmans^168^), the technical performance of most of the aforementioned DBT systems is compared across several key metrics. These metrics include the modulation transfer function (MTF), noise power spectrum (NPS), DQE, artifact spread function (resolution in the z-direction), signal difference to noise ratio (SDNR), mean glandular dose (MGD), threshold diameter for detecting microcalcification specks, and threshold diameter for identifying non-spiculated masses.

A brief summary of some of the features of the tomosynthesis systems currently available for body imaging (e.g., chest, knee, and leg) is provided in Table 2.

**Table 2:** Some characteristics of present-day body tomosynthesis systems.

Unit	Tomo angle (deg)	# views	Pixel pitch (μm)	2 × 2 binning	Detector	Scan time (s)
Knee study						
Carestream HS^a^	40	41	278	Yes	CsI-a-Si	5.1
Carestream HR^a^	40	41	139	No	CsI-a-Si	10.2
Fujifilm	40	40	150	No	CsI-a-Si	8
GE	40	40	200	No	CsI-a-Si	8
Shimadzu	40	74	150	Yes	CsI-a-Si	5
Chest study						
Carestream HS	30	61	278	Yes	CsI-a-Si	7.6
Carestream HR	30	61	139	No	CsI-a-Si	15.2
Fujifilm	27	60	150	No	CsI-a-Si	12
GE	30	60	200	No	CsI-a-Si	11.3
Shimadzu	40	74	150	Yes	CsI-a-Si	5

a HS, standard mode; HR, high-resolution mode.

## 9 X-Ray Tubes

The majority of tomosynthesis systems used for imaging both the breast and body are designed to perform conventional digital radiographic (2D) imaging as well as tomosynthesis (3D) imaging. Consequently, these systems typically employ the same x-ray sources (x-ray tube and beam filter) and detectors for both imaging modes. However, there are some notable exceptions. For instance, the Hologic DBT systems use different x-ray beam filters for 2D and 3D imaging—a tungsten target with a rhodium filter for 2D imaging and a tungsten target with an aluminum filter for 3D imaging (Marshall and Bosmans^168^). This configuration results in a higher effective energy and a more transmissive beam for 3D imaging, enabling shorter exposure pulses for the 15 projection views acquired with a continuously moving x-ray tube. This design helps reduce motion blur caused by the x-ray tube.

To further mitigate x-ray tube blur, some systems, such as the GE Pristina, employ a step-and-shoot acquisition method where the x-ray tube stops for each exposure. However, the acceleration and deceleration involved in the step-and-shoot method can lead to a longer overall acquisition time compared with continuous tube motion with pulsed exposure, potentially increasing the risk of patient (breast) motion blur.

Four alternative x-ray source designs have been investigated to minimize both x-ray tube motion and patient motion degradation of spatial resolution in tomosynthesis images.

### 9.1 Carbon Nanotube X-Ray Sources

This approach involves an array of multiple distributed stationary carbon nanotube (CNT) x-ray sources. Research on this technology has shown promise for both DBT (Qian^169^) and chest tomosynthesis (Burks^170^). CNT sources allow for rapid switching and precise control, reducing motion artifacts and potentially improving image quality.

### 9.2 Multiple X-Ray Tubes

This approach uses multiple independent x-ray sources to reduce the motion needed to acquire a specific angular range. Both conventional and cold-cathode x-ray sources have been proposed and are currently being developed into prototype products, including the AIXscan using conventional sources and the Nanox using cold-cathode sources.

### 9.3 Multi-X-Ray Source X-Array

This design features an array of grid-controlled tungsten filament cathodes linearly distributed around a set of molybdenum anodes that rotate about a common axis. All these components are housed within a vacuum envelope (Becker^171^). This innovative setup allows for synchronized rotation and x-ray emission, minimizing both tube and patient motion blur, and enhancing spatial resolution.

### 9.4 Flying Focal Spot Source with Continuous X-Ray Tube Motion

This approach uses the flying focal spot technology that was originally developed for CT scanners. This technology steers the electrons striking the target of the x-ray tube during projection view acquisition in such a way that the position of the effective focal spot remains stationary during each exposure. This, as previously mentioned, is used in the new Siemens B.brilliant DBT system, allowing a scan time of about 5 s, helping to reduce and minimize patient motion artifacts (Kappler^172^).

## 10 Promising New Applications and Developments in Tomosynthesis Imaging

Researchers are exploring new applications of tomosynthesis, such as contrast-enhanced imaging and multimodal systems that combine tomosynthesis with ultrasound, nuclear medicine, and optical imaging. These advancements aim to improve diagnostic accuracy and expand the clinical utility of tomosynthesis.

### 10.1 Contrast-Enhanced Applications

Digital subtraction angiographic applications of tomosynthesis, initially developed using image intensifier detectors, are now being revisited with modern flat-panel detectors. For breast imaging, dual-energy contrast-enhanced spectral 2D mammography (CESM) has been commercialized. This technique enhances the visibility of blood vessels and masses in the breast. However, superposition in 2D CE imaging has the potential for the creation of pseudomasses and the obscuring of real masses. These issues can be addressed by using tomosynthesis. Research in CE DBT was first described in 2004 by Tao Wu et al. (AAPM meeting 2004).^173^ Subsequently, CE DBT was tested in clinical trials by Maidment’s research group (Chen^174^) where the CE-DBT images were shown to be concordant with contrast-enhanced breast MRI. Furthermore, the spatial resolution of CE DBT is significantly better than that of MRI, and similar to MRI, the dynamic curves of contrast in the masses can be measured. The latter would involve additional x-ray dose.

### 10.2 Multimodal Breast Imaging Systems

Combining tomosynthesis with other imaging modalities, such as automated 3D ultrasound and molecular breast imaging (MBI), offers enhanced detection and characterization of breast lesions. These systems ensure a one-to-one correspondence between masses observed in different modalities, solving the issue of non-corresponding lesions due to different imaging geometries.

#### 10.2.1 Combined tomosynthesis and automated ultrasound imaging

Scientists at the University of Michigan and GE Global Research collaborated to develop an advanced combined x-ray tomosynthesis and automated 3D ultrasound breast imaging system (Kapur,^175^ Carson,^176^ Booi,^177^ and Sinha^178^). This innovative system leverages ultrasound imaging to supplement x-ray tomosynthesis, enabling the distinction between cysts and tumors and providing additional information for characterizing lesions.

**Fig. 3:**
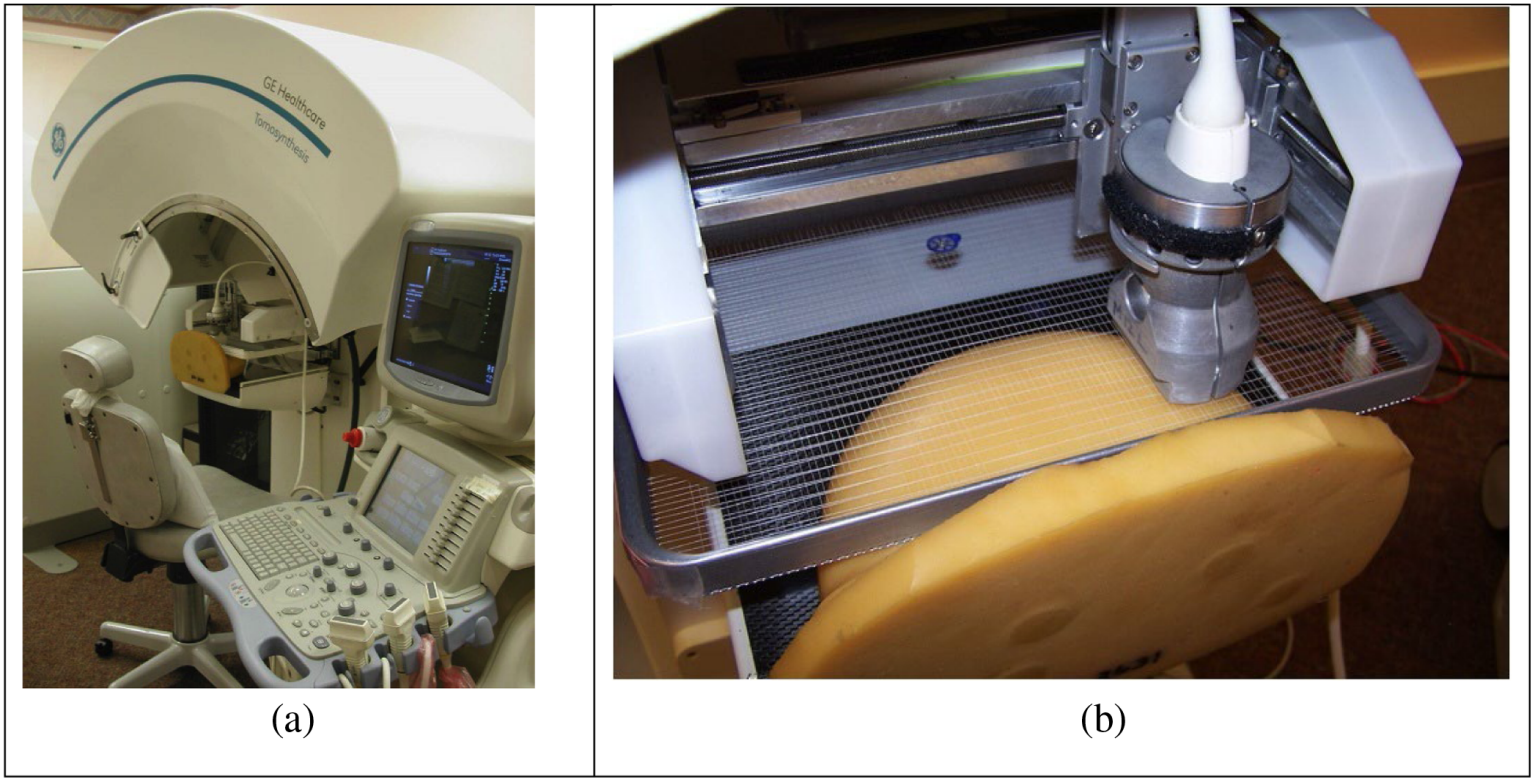
Combined tomosynthesis and automated ultrasound breast imaging system developed at the University of Michigan is shown in the ultrasound image acquisition position while scanning a breast-simulating phantom (a). A closeup of the ultrasound transducer scanning mechanism and dual-modality mesh paddle (b). This mechanism is flipped up out-of-view for DBT acquisition.

The combined system comprises a GE GEN II prototype tomosynthesis unit equipped with a dual-modality (x-ray and ultrasound) mesh compression paddle and an ultrasound transducer translator (See Fig. 3). The GEN II tomosynthesis system features a CsI-aSi flat-panel x-ray detector like the one used in the commercial GE Essential system. The x-ray tube moves in an arc in a step-and-shoot mode, with a tomosynthesis angle of 60 deg and 21-angle increments in 7.5 s. The patient remains seated throughout the dual-modality procedure.

The breast compression paddle, made of a nylon mesh, is compatible with both x-ray and ultrasound imaging. During tomosynthesis acquisition, the ultrasound transducer is out of view. For ultrasound imaging, the transducer is rotated into position, and acoustic coupling gel is applied between the transducer, the paddle, and the breast. A GE Logiq 9 ultrasound system with an M12L transducer operated at 10 MHz is used. The transducer scans the breast in an x-y raster mode, producing an image every 0.8 mm. Software was developed to automatically register volumes of interest in both tomosynthesis and ultrasound images (Goodsitt^179^).

The University of Michigan team has been developing a photoacoustic tomography system (Wang^180^) that can be combined with the DBT and automated ultrasound imaging unit. In photoacoustic tomography, short pulses (e.g., <25  nsec) of near-infrared laser light (e.g., 720 to 900 nm) are directed at the breast, heating the inner tissues, and causing thermo-expansion, which emits ultrasonic waves. These waves are detected with a large 2D receiving ultrasound transducer array, and a backprojection reconstruction algorithm produces an image of the optical absorption in the breast. In this system, the laser light is coupled to the compression paddle above the breast with a fiber optic array, and the ultrasound transducer receiving array is placed beneath the breast. The use of two wavelengths of laser light in spectroscopic photoacoustic tomography allows for functional imaging by distinguishing between oxygenated and deoxygenated hemoglobin.

One challenge with combined x-ray tomosynthesis and automated ultrasound breast imaging systems is limited ultrasound breast volume coverage, especially at the periphery due to inadequate acoustic coupling. At the University of Michigan, one solution investigated was the use of a gel retainment dam (Li^181^), which increased the area of the breast in contact with the compression paddle by an average of 14%. Another solution implemented in a different combined “FUSION-X-US-II” prototype system involves placing a special air cushion between the breast support plate and a dual-modality gauze compression paddle (Schäfgen^182^). After the breast is compressed, the air cushion is inflated, raising the peripheral part of the breast toward the gauze paddle. This combined system includes an ACUSON S2000 automated breast volume scanner with a large linear array transducer that scans the entire breast in one sweep and a Siemens Healthcare MAMMOMAT Inspiration DBT system. In a study involving 30 healthy women, the authors estimated that ultrasound coverage of the breast was 90.8%.

Finally, another method to maximize breast volume coverage in the ultrasound component of combined DBT and automated breast ultrasound (ABUS) imaging is to perform the ABUS with the patient in the conventional supine orientation. Deformable mapping is then used to correlate lesions in the ABUS and DBT x-ray images. This method was recently developed at the University of Michigan (Green^183^^,^^184^). In a preliminary patient study, the use of external fiducial markers glued to the patient’s breast helped improve registration results. For craniocaudal (CC) DBT mapped to ABUS in seven patient data sets, the mean distances between the centers of mass (dCOM) of nine corresponding lesions using six markers were 14.9±6.8  mm. For mediolateral oblique DBT mapped to ABUS, the mean dCOM was 13.7±6.8  mm for eight corresponding lesions using six markers (Green^184^).

#### 10.2.2 Combined x-ray tomosynthesis and nuclear medicine imaging

Mark Williams and his team at the University of Virginia have developed a system that integrates x-ray tomosynthesis with molecular breast imaging (MBI) tomosynthesis (Williams^185^ and Patel^186^). The system utilizes a GE SenoClaire DBT unit combined with a gamma camera specifically designed for breast imaging. MBI involves the administration of a radiotracer, such as technetium-99m sestamibi, which preferentially accumulates in malignant breast tissue due to its increased metabolic activity. The gamma camera detects the emitted gamma rays from the radiotracer, providing functional images that highlight areas of increased radiotracer uptake, which often correspond to malignant lesions.

The integration of these two imaging modalities is achieved through a full isocentric scanning motion, with both the x-ray tube and the gamma camera rotating about a central axis. This setup ensures that both anatomical and functional images are acquired from the same geometrical perspective, facilitating accurate image fusion and correlation. The synchronized rotation allows for the simultaneous acquisition of DBT and MBI images, minimizing patient movement and ensuring precise alignment between the two datasets.

Clinical studies have demonstrated that the combined DBT-MBI system significantly enhances the detection of breast cancer, particularly in women with dense breast tissue. Dense breast tissue can obscure lesions on traditional mammograms, reducing the sensitivity of cancer detection. The functional information provided by MBI complements the anatomical detail from DBT, improving overall diagnostic accuracy. The high-resolution tomosynthesis images enable precise localization and characterization of lesions, whereas the MBI images provide additional information about the metabolic activity and potential malignancy of the detected lesions. This dual-modality approach enhances the specificity of breast cancer diagnosis, reducing the number of false positives and unnecessary biopsies.

Moreover, the combined system has shown promise in detecting smaller and more aggressive cancers that might be missed by conventional imaging methods. The increased sensitivity of the combined DBT and MBI approach allows for the identification of early-stage cancers, which is crucial for improving treatment outcomes and patient survival rates.

Significant improvements in the image quality of DBT within combined DBT and molecular breast tomosynthesis systems have been reported (Patel^186^). These enhancements, particularly in contrast and SDNR, were achieved without increasing the breast dose. This was accomplished through the incorporation of a prototype 2D cellular-focused reciprocating anti-scatter grid positioned near the x-ray detector.

Separately, researchers at the University of Pennsylvania have developed a combined DBT and positron emission tomography (PET) imaging system. The system consists of a custom-built DBT system in which the x-ray source and detector move through a more complex scanning motion to improve the 3D reconstruction of the breast and a custom-built PET camera which is placed adjacent to the breast to maximize gamma-ray collection efficiency and reduce radiation dose. The PET acquisition and reconstruction, like DBT, is based on a limited angle technique as the PET detectors do not completely encircle the breast. Initial results with phantoms are encouraging, and a clinical trial is ongoing.

Future advancements in both x-ray and molecular imaging technologies, such as larger fields of view and shorter imaging times, may provide additional benefits for dual-modality imaging, further enhancing diagnostic capabilities and patient outcomes.

#### 10.2.3 Combined tomosynthesis and optical imaging

Researchers at Massachusetts General Hospital, Tufts University, and Northeastern University have developed an innovative system that combines x-ray tomosynthesis with diffuse optical tomography (DOT) for breast imaging (Fang^187^^,^^188^). DOT is a functional imaging technique that uses near-infrared lasers to probe tissues, with the optical absorption and scattering of the laser light providing information related to physiological parameters such as concentrations of hemoglobin, oxygenated hemoglobin, water, and lipids. A major drawback of DOT alone is its poor spatial resolution, which is significantly improved when combined with the anatomical detail provided by x-ray tomosynthesis.

The combined system utilizes a GE DS clinical prototype tomosynthesis unit that captures 15 projections over a 45-deg tomosynthesis angle, with a pixel size of 0.1 mm and a reconstructed slice thickness of 1 mm. The optical system includes two continuous-wave laser systems: one with three lasers (685, 810, and 830 nm) directed by a fast Galvo scanner, and the other with 26 lasers (13 at 685 nm and 13 at 830 nm) for continuous monitoring. The laser source probes are placed in a cassette above the x-ray detector, whereas the detector probes are in an optically transparent dual-modality compression paddle.

In a study involving 125 subjects and 189 breasts, it was found that malignant tumors had significantly higher total hemoglobin concentration compared with fibroglandular tissue, providing a functional imaging marker that could help distinguish between benign and malignant lesions (Fang 2011^188^).

In a later publication, Zimmerman^189^ described a novel DOT system developed by their research group that enables simultaneous DBT and DOT acquisition. To reduce artifacts in the DBT images caused by x-ray attenuation from the DOT system, they utilized plastic rather than glass fiber optics. The system integrates 96 continuous-wave and 24 frequency-domain source locations, as well as 32 continuous-wave and 20 frequency-domain detection locations into low-profile plastic plates. These plates can easily mate with the DBT compression paddle and x-ray detector cover. The system is dynamic, with a current DOT frame acquisition duration of 3 s, which can potentially be reduced to 1 s. This capability allows the assessment of the hemodynamic response of the breast tissue at both partial and full mammographic compression during an exam. Previous studies with their stand-alone DOT system indicated a statistically significant difference in the pressure response of tumor tissue compared with healthy tissue (Carp^190^).

Recently, a group in South Korea developed a system that fused DBT and DOT images (Chae^191^). These images were acquired separately using a commercial GE SenoClaire DBT system (GE Healthcare, Waukesha, United States) and a prototype DOT system developed at the Korea Electrotechnology Research Institute. Two readers evaluated the DBT and DOT images separately, as well as the fused images. The areas under the receiver operating characteristic curves for the readers improved significantly for the fused images, and the interobserver agreement was highest for the fused images.

## 11 Historical Evolution of Tomosynthesis Timeline

**Fig. 4:**
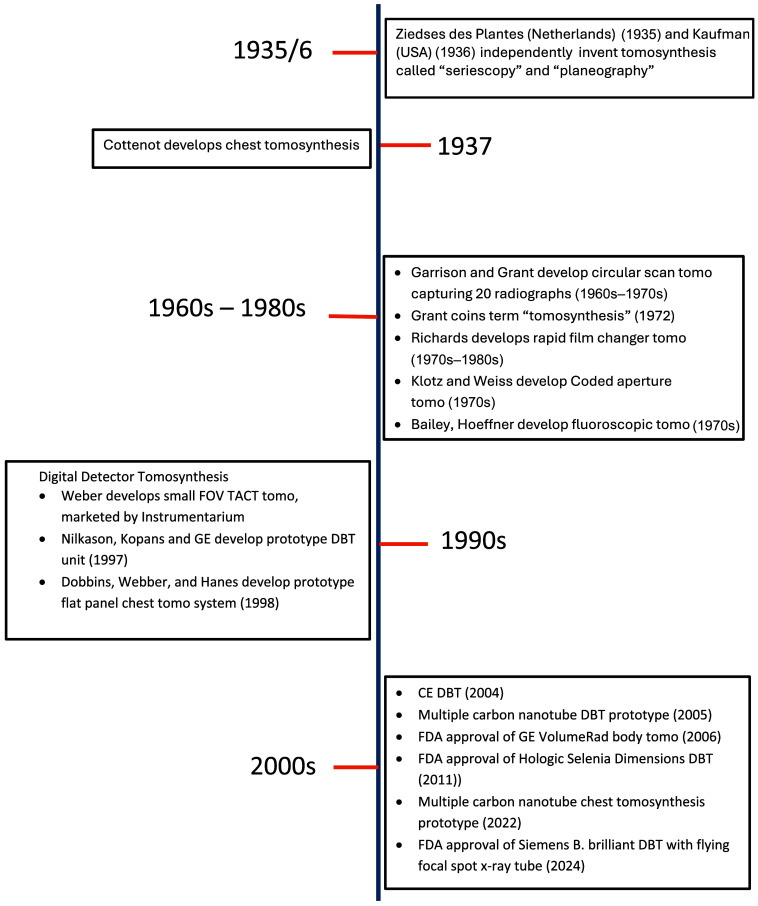
Historical evolution of tomosynthesis timeline.

The historical evolution of tomosynthesis imaging that is described in this review paper is summarized in the timeline shown in Fig. 4. Most of the early systems were prototypes. The first commercial system to our knowledge was Richard’s Dynatome rapid film changers unit, which was marketed in the 1970s and 1980s. Instrumentarium was the first company to sell a small field-of-view DBT system, which was FDA-approved in 2000. Hologic was the first company to receive FDA approval for a full field-of-view DBT system. That approval in 2011 was for their Selenia Dimensions system. Subsequently, all of the DBT units listed in Table 1 have received FDA and/or CE Mark (Conformite Europeenne) approval. GE was the first company to receive FDA approval for their VolumeRad whole-body tomosynthesis system, in 2006. Subsequently, all of the other companies listed in Table 2 plus some others received FDA and/or CE Mark approval.

Tomosynthesis is often described as limited-angle CT. Most present-day implementations of tomosynthesis involve limited angle cone beam CT using flat-panel digital detectors. The beginnings of tomosynthesis (in the 1930s) predate that of CT (in the 1960s) because tomosynthesis could be accomplished without the need for computers using film detectors and optical displays. The first commercial CT system was manufactured by EMI and was exhibited at the 1972 Radiological Sciences in North America meeting. This was soon followed by the development and commercialization of CT scanners by many other manufacturers in the 1970s, 1980s, and beyond [e.g., AS&E, Varian, OmniMed, Imatron, Ohio Nuclear, Pfizer, Siemens, GE, Picker, Philips, Hitachi, Toshiba, Elscint, and Compagnie Générale de Radiologie (CGR)].^192^ Thus, significant commercialization of CT predates that of tomosynthesis by about 20 to 30 years. Many advances in CT technology have been and continue to be applied to tomosynthesis, especially in the area of image reconstruction (e.g., filtered backprojection, iterative, model-based, and deep learning) and most recently in flying focal spot x-ray tube design.

## 12 Future of Tomosynthesis

Tomosynthesis continues to evolve, and it is likely that with technical advances, the image quality will improve, the patient dose will decrease, the image display will progress, and dual-modality applications will expand. Some of the technical advances will likely include

• better multiple source x-ray tubes such as carbon nanotubes with much greater x-ray flux, smaller focal spots, and greater longevity
• new configurations for tomosynthesis acquisitions in different geometries than linear
• higher DQE detectors such as those that are being developed using perovskite
• improved image reconstruction algorithms
• virtual reality displays

Recently, photon counting detectors in CT have resulted in significant advances in tissue discrimination, spatial resolution, and dose reduction. Previously, DBT prototypes using linear photon counting detectors and scanning slit acquisitions were developed and showed promise. Two examples were the XCounter XC Mommo-3T which used 48 noble gas-filled linear strip detectors^193^ and the Sectra/Philips system based on the MicroDose Mammography SI unit which used 21 silicon strip line detectors.^194^ Neither prototype was a commercial success, perhaps due to the mechanical complexities, longer scan times, and limited tomosynthesis angles associated with the multiple scanning slit acquisitions. In the future, large-area photon counting detectors may be developed that would eliminate the need for scanning slits and could be used in clinically practicable DBT systems as well as body tomosynthesis systems.

Finally, artificial intelligence has a great potential for improving outcomes in tomosynthesis. In a recent New Horizons paper that was published *in Radiographics*, Goldberg et al.^195^ discuss many areas in which AI has been applied in DBT. Some of these include “non-inferior or improved sensitivity and accuracy for breast cancer detection, decreased workload, decreased recall rates, localization and classification of abnormal imaging findings, increased conspicuity of suspicious lesions, reduced radiation dose, breast cancer risk assessment, and minimization of imaging artifacts.”

## 13 Conclusion

Tomosynthesis has had a fascinating and dynamic history, beginning in 1935 and continuing to evolve into the sophisticated imaging technology used today. This paper on the history of tomosynthesis draws on several meticulously documented sources, providing a comprehensive overview of its development.

The foundation of this paper includes insights from two extensive reviews by Dobbins^196^ and Godfrey.^167^ These reviews provide a detailed chronology of the advancements in tomosynthesis, from its early conceptual stages to its modern applications. The work of Dobbins and Godfrey highlights the technological innovations, clinical trials, and regulatory milestones that have shaped the field.

The book *From the Watching of Shadows: The Origins of Radiological Tomography* by Webb^4^ offers an in-depth exploration of the early days of tomographic imaging. Webb’s meticulous documentation of the pioneering efforts and breakthroughs in radiological tomography provides a historical context that is crucial for understanding the evolution of tomosynthesis.

For those specifically interested in breast tomosynthesis, Sechopoulos’ two-part review is invaluable.^197^^,^^198^ Part I covers the image acquisition process, detailing the technical aspects of how tomosynthesis images are captured, including the geometry of image acquisition and the types of detectors used.^197^ Part II delves into image reconstruction, processing, and analysis, as well as advanced applications of breast tomosynthesis, such as its use in screening and diagnostic contexts, and its potential in contrast-enhanced tomosynthesis.^198^

Furthermore, the recent paper by Marshall and Bosmans^168^ provides a thorough performance evaluation of DBT systems. This paper compares various systems based on critical parameters such as MTF, NPS, DQE, artifact spread function, SDNR, MGD, and the thresholds for detecting microcalcifications and non-spiculated masses. Their analysis offers valuable insights into the strengths and limitations of current tomosynthesis technologies and sets the stage for future improvements.

The reader is encouraged to refer to these foundational references for a more detailed understanding of the history and development of tomosynthesis. These sources, along with others listed in the reference section, provide a wealth of information and deeper insights into the technological advancements, clinical applications, and ongoing research that continue to drive the field of tomosynthesis forward.

## Data Availability

Data sharing is not applicable to this article, as no new data were created or analyzed.
